# A Novel Way to Relate Ontology Classes

**DOI:** 10.1155/2015/724196

**Published:** 2015-04-23

**Authors:** Ami T. Choksi, Devesh C. Jinwala

**Affiliations:** Computer Engineering Department, Sardar Vallabhbhai National Institute of Technology, Surat, Gujarat 395007, India

## Abstract

The existing ontologies in the semantic web typically have anonymous union and intersection classes. The anonymous classes are limited in scope and may not be part of the whole inference process. The tools, namely, the pellet, the jena, and the protégé, interpret collection classes as (a) equivalent/subclasses of union class and (b) superclasses of intersection class. As a result, there is a possibility that the tools will produce error prone inference results for relations, namely, sub-, union, intersection, equivalent relations, and those dependent on these relations, namely, complement. To verify whether a class is complement of other involves utilization of sub- and equivalent relations. Motivated by the same, we (i) refine the test data set of the conference ontology by adding named, union, and intersection classes and (ii) propose a match algorithm to (a) calculate corrected subclasses list, (b) correctly relate intersection and union classes with their collection classes, and (c) match union, intersection, sub-, complement, and equivalent classes in a proper sequence, to avoid error prone match results. We compare the results of our algorithms with those of a candidate reasoner, namely, the pellet reasoner. To the best of our knowledge, ours is a unique attempt in establishing a novel way to relate ontology classes.

## 1. Introduction

The semantic web [1–4] enables computers to understand the meaning or semantics of the information available on the web. The semantics have greatly benefited the research of integration, search, and analysis of data. The semantic web is a vision of “web of linked data.” The Linked data are represented by structured or semistructured ontologies and are described by languages, namely, Resource Description Framework (RDF) [5] and web ontology language (OWL) [6–8]. An ontology is a formal specification of the conceptualization. The concept is represented using classes and properties and relations among them. The classes are of different types, namely, subclass, superclass, intersection class, union class, and complement class. The properties are mainly of two types, namely, data property and object property.

The vendors publish resource ontologies in the semantic web repositories. The user, who wants to discover ontologies, specifies the description of the required one. The resources in repositories are called advertised resources and user's required resource description is named as requested resource. The semantic description of resources is preferred over syntactic description, because of being more expressive, sharable, and maintainable [9–11].

The resource discovery is a process of finding the advertised resource(s), those which are relevant to the request [12–15]. The unlimited size of the semantic web makes the resource discovery a challenging task in the semantic web [16].

The existing ontologies in literature typically use* anonymous* union and intersection classes. The anonymous classes are limited in scope. They may not be part of the whole inference process by ontology reasoners [17]. Therefore, there is a need to use* named* union and intersection classes in the ontology.

As we observe, the inference based on the union and the intersection classes are partially available in the tools, namely, the jena semantic framework [18, 19], the Pellet reasoner [20], and the protégé editor [21, 22]. The collection classes are interpreted as (i) either subclasses or superclasses of union and intersection classes, respectively, by the tools, namely, the jena and the Pellet and (ii) equivalent classes of union class by the ontology editor, namely, the protégé. Both of the interpretations are conceptually error prone, as (a) if a class is going to be interpreted as the equivalent/superclass, why should we make it the union class? and (b) if a class is going to be interpreted as a subclass, why should we make it the intersection class? In the example subsection we discuss how the above interpretations are conceptually error prone using an illustration. We believe, unless we specifically mention to treat the class as equivalent, subclass, or superclass, they should be interpreted in their own original form ONLY. Therefore, a need arises to establish a proper relation between union and intersection classes and their collection classes. This indeed will affect the sub- and equivalent classes list of any class in ontology, wherein union and intersection classes are part of the ontology, which we believe. This too will affect the complement class list as it is dependent on sub- and equivalent classes internally. Therefore, there is a need to calculate correct sub-, equivalent, and complement classes list of all the classes in the ontology. We believe it can be achieved through proper sequence of matching above classes in a match algorithm.

We, therefore, attempt to refine our test application data set of the conference management system ontology [23] by adding the* named*, union, and intersection classes. We propose a match algorithm to (a) relate collection classes to their union and intersection classes in their original form only, (b) refine the subclasses list of any class of the ontology wherein* named* union and intersection classes are part of the ontology tree, to rectify inferring process, and (c) match complement, union, intersection, and equivalent classes in a proper sequence to avoid error prone match results.

We compare the results of our algorithm with the candidate reasoner, namely, the Pellet. To the best of our knowledge, ours is a unique attempt in rectifying the inferring process for* named*, union, and intersection classes.

Our contributions in the paper are as follows.

- We refine the conference ontology [23] of our test application of the conference management system by adding* named*, intersection, and union classes.
- We propose an algorithm to
  - correctly relate* named*, intersection, and union classes to their collection classes,
  - refine the subclasses list of any class of the ontology wherein* named* union and intersection classes are part of the ontology tree, to rectify inferring process,
  - match ontology classes in a proper sequence for sub-, complement, equivalent, union, and intersection classes.
- We compare the results of our algorithm with those of the candidate reasoner, namely, the Pellet.

The organization of the paper includes 7 sections. The first one we have already described, that is, the Introduction. The subsection of Introduction is the example, wherein we discuss the error prone interpretations of tools, namely, the protégé, the jena and the Pellet. In Section 2, we present the theoretical background, in Section 3 we describe the problem statement, in Section 4, we present our proposed algorithm, in Section 5, we describe methodology of implementation, in Section 6, we describe performance results and analysis, and in Section 7, we present the conclusion and future work.

### 1.1. The Example

An illustration shows the error prone results achieved in interpreting union class as superclass and intersection class as subclass of collection classes.

The Student and Employee are two classes with properties and individuals as follows:

1. Student (class) is as follows:
  - Properties = {name, rollno, branch}.
  - Individuals = {Ami, Abhay, Aayushi, Ashish}.
2. Employee (class) is as follows:
  - Properties = {name, EmployeeID, department},
  - Individuals = {Ami, Ashish, Tushar}.
3. Let us take the intersection of the classes. That is, all the individuals and properties which are common in both* Student and Employee*. Let us name intersection class as* StudentNEmployee*. The properties and individuals of the* StudentNEmployee* class should be as follows.
4. StudentNEmployee (intersection class) is as follows:
  - Properties = {name}.
  - Individuals = {Ami, Ashish}.
5. Let us take the union of the classes now. That is, all the individuals and properties which are either* Student or Employee* as well as those who are both* Student and Employee*. Let us name union class as* StudentOrEmployee*. The properties and individuals of the* StudentOrEmployee* class should be as follows.
6. StudentOrEmployee (union class) is as follows:
  - Properties = {name, rollno, EmployeeID, branch, department}.
  - Individuals = {Ami, Abhay, Aayushi, Ashish, Tushar}.
7. Let us consider MechanicalStudent as the subclass of* Student class*. The properties and individuals of the class are as follows.
8. MechanicalStudent (subclass) is as follows:
  (i) Properties = {**name**,** rollno**,** branch**, appronid}.
  (ii) Individuals = {Ashish}.

The properties in bold are inherited from* Student* class, and they too are part of* MechanicalStudent*.

From the definition of subclass [24], one can say subclass has more properties/characteristics than its base class. From the definition of equivalent class, one can say the classes should have equal individuals. If we interpret

- *collection classes as sub classes of union class*, the* Student* class should have more properties than the* StudentOrEmployee* class. According to the 1st and 4th points, we can say, the inferred fact is error prone;
- *intersection class as the sub class of the collection classes*, the* StudentNEmployee* class should have more properties than* Student* class. From the 1st and 3rd points, we can say, the inferred fact is error prone;
- *collection classes as equivalent classes of union class*, the* Student* class should have same individuals as the* StudentOrEmployee* class. From the 1st and 4th points, we can say, the inferred fact is error prone.

The error prone interpretations of union and intersection classes as super classes or equivalent classes/subclasses of collection classes, in turn, will create error prone sub- and equivalent classes lists. The match process, wherein the sub- and/or equivalent classes are involved, will also produce error prone match results.

## 2. Theoretical Background and Literature Survey

The resource discovery in the semantic web consists of ontology resources. The resource ontology is described using individuals, classes, attributes/properties, relations, restrictions, rules, and axioms like components.

Few terminologies for understanding matching algorithms are defined as follows.

### 2.1. Terminologies

The following terms are useful in understanding the paper.

(i) Subclass: a class C1 is defined as a subclass of class C2, which means the set of individuals of C1 should be a subset of the individuals of C2.
(ii) Superclass: a class C1 is defined as a superclass of class C2, which means the set of individuals of C2 should be a subset of the individuals of C1.
(iii) Complement class: a class C1 is defined as complement of class C2, if C1 contains exactly those individuals that do not belong to C2. ComplementOf in OWL is the logical negation. It is the same as if the NOT operator of set theory [25] is applied to classes.
(iv) Equivalent class: a class is said to be equivalent class if they have the same individuals.
(v) Union class: a union class is having all individuals of its collection classes at least once. It is the same as if the OR operator of set theory applied to classes. The collection classes are said to be operands too.
(vi) Intersection class: an intersection class is having only those individuals which are common in all of its collection classes. It is the same as if the AND operator of set theory is applied to classes.
(vii) Equivalent class: if class *A* and class *B* are equivalent classes. They have exactly the same set of individuals.
(viii) Anonymous class: anonymous class is a class without a name/identifier.
(ix) Resource discovery: resource discovery is a process of finding relevant resource for the application under consideration.
(x) Precision ratio: the ratio of the number of relevant retrieved resources to the total number of resources retrieved is a precision ratio [26]. Consider
  (1) Precision  Ratio =relevant  resources∩retrieved  resourcesretrieved  resources.
(xi) Recall ratio: the ratio of the number of relevant retrieved resources to the number of relevant resources is a recall ratio [26]. Consider
  (2) Recall  Ratio =relevant  resources∩retrieved  resourcesrelevant  resources.
(xii) Matching: matching is the process of finding correspondences between semantically related entities of different resources. Advertised resource is considered to be *A* and requested resource is considered to be *R*. Properties are considered to have *P*1, *P*2,…, *Pn* for *A* and *P*1, *P*2,…, *Pm* for *R*. Matching [27–29] can be exact, subsume, plug-in, or no-match. The terminologies used in this work are as follows.
  (a) Exact match: if all the properties of the advertised resource match with those of the requested one, it is called exact match. Consider
    (3) A(PROPERTIES)≡R(PROPERTIES) ⟶APi≡RPi.
  (b) Subsume match: if advertised resource properties are less than those of requested resource, it is called subsume match. Consider
    (4) A(PROPERTIES)≤R(PROPERTIES) ⟶APi⊆RPi.
  (c) Plug-in match: if advertised resource properties are greater than those of the requested resource, it is called plug-in match. Consider
    (5) A(PROPERTIES)≥R(PROPERTIES) ⟶APi⊆RPi.
  (d) No match: if advertised resource properties and requested resource properties do not have any of the above relations, it is called No match. Finding match can be done using semantic similarity [30–33], semantic distance [34, 35], and feature matching.
(xiii) Semantic similarity: a similarity function is defined as a real valued function as follows:
  (6) $$\begin{array}{c} \text{sim}\left(x,y\right):CXC⟶\left[0,1\right]. \end{array}$$
 On a set measuring the degree of similarity between *x* and *y*, where *C* is the set of well-formed concepts and where sim(*x*, *y*) measure the degree to which y is similar to x, sim(*x*, *y*) = 1 means fully similar, namely, the case *x* = *y*; sim(*x*, *y*) = 0 means nothing between two concepts. Consider
  (7) Simx,y=pAx⋂BxAx +1−pAx⋂BxAy.
(xiv) Structural matching: structural matching [36] involves matching of parameters, namely, ancestors, descendants, leaves, adcacents, ascopath, dsipath, siblings or subclasses list, and similarity contribution.
(xv) Ontology alignment: it is a process of establishing a collection of binary relation between the vocabularies of two ontologies. Ontology alignment is applied to the same or related domain ontologies. Ontology alignment [37–39] techniques are broadly categorized into terminological, structural, extensional, and semantic techniques.
(xvi) Reasoner: a reasoner or inference engine is used to derive the facts which are true but syntactically difficult to achieve. A reasoner can improve efficiency of matching.

### 2.2. Related Work

A resource discovery is a matching process of requested and advertised resources. Resources in the semantic web are ontologies. The classes and properties of two ontologies are matched using linguistic, string distances, structural, or combined techniques [37–40].

We observe, in the ontologies available in the literature, the union and intersection classes are* typically* used as* anonymous* classes. The scope of anonymous classes is limited; they may not participate in whole inference process of a reasoner [17]. Therefore, there is a need for* named* union and intersection classes.

The reasoners, namely, Pellet, Fact++, RacerPro, Hermit, and so forth [41] are used to infer the hidden information from the ontologies. The* Pellet* is a better reasoner from points of view of the reasoning characteristics, practical usability, and performance. The comparison of reasoners is discussed in [20, 41].

Similar to the reasoner, the jena semantic framework too has query engine in it. Therefore, we are motivated to check the inference support of tools, namely, jena and Pellet for the* named* union and intersection classes. The tools have partial support for the* named* union and intersection classes [19]. The tools support inference for individuals of union and intersection classes. Let us compare query engines of the tools, namely, Pellet and jena [42], as shown in Table 1. The table shows query engine of Pellet is better than that of jena.

Though the Pellet's query engine is better than that of jena, yet they both interpret collection classes of union and intersection as subclasses and superclasses, respectively.

The designers create ontologies using ontology editors. The characteristics of support for Java, open source, and support for creating simple as well as complex ontologies make the protégé editor performs better than other existing editors, namely, Apollo, OntoStudio, Swoop, and TopBraid. The comparison of ontology editors is discussed in [21, 22]. We need to check whether* named* union and intersection classes are added properly or not.

From above points of discussion, we use Pellet reasoner and protégé editor as our candidate tools for our work. The comparison in Table 2 shows how the tools, namely, protégé editor and Pellet reasoner interpret the union and the intersection classes.

As discussed in The Example subsection, such interpretation may lead to inference of error prone subclasses and equivalent classes list. In turn, the error prone list will affect other operations which use the sub and equivalent classes list. For example, complement class matching in turn uses sub classes as explained in [43], and ontology structure matching algorithms too uses sub classes list as one of the matching features [36].

Therefore, there is a need to have match algorithm to establish correct interpretations of* named* union and intersection classes, as well as proper relation with their collection classes. To avoid error prone match results of the algorithm involving sub-, union, intersection, complement, and equivalent classes, there is a need to decide a proper sequence of match.

## 3. The Problem Statement

**Figure 1:**
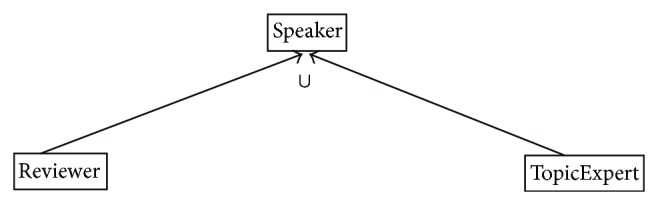
Union class example.

**Figure 2:**
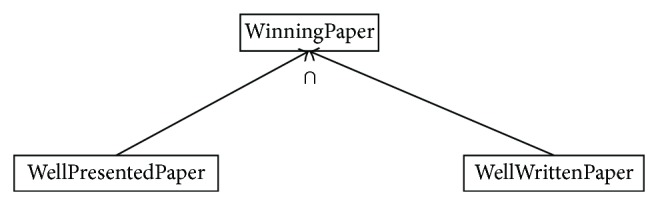
Union class example.

Anonymous union and intersection classes of an ontology may not take part in the whole inference process of a reasoner. Treating union class as super/equivalent class of its collection classes and intersection class as subclass of its collection classes produces error prone match results. They produce error prone subclass and equivalent classes too. This will affect the matching process wherein subclasses list or equivalent classes list are used. Action is needed to (a) add named union and intersection classes in the ontology, (b) interpret union and intersection classes in their original form, (c) relate union and intersection classes properly to their collection classes, and (d) avoid error prone match results of union, intersection, sub-, super- and complement classes. One can easily understand the problem statement with respect to the examples of the conference management system, as shown in Figures 1 and 2.

## 4. Our Proposed Algorithm

The ontology resource discovery is a process of matching requested and advertised resources. As part of the ontology resource discovery, we need to match the classes of ontology. Therefore, the scope of our work is ontology matching/resource discovery.

We propose a match algorithm that operates on the* named* union and intersection classes. We are aiming to

1. interpret the named union and intersection classes in their original form,
2. relate the union and intersection classes properly to their collection classes,
3. create a proper match sequence to avoid error prone results for union, intersection, sub-, complement, and equivalent classes.

We implement our work using two algorithms, (i) subclasses calculation algorithm and (ii) a match algorithm. In this section, we describe the algorithms which we employ and their logic of working and analyze their complexities.

### 4.1. The Logic

The algorithms work on the following logic principles.

(1) The subclass calculation algorithm is as follows.
  (a) In ontology, we have *C* as union class of *A* and *B* collection classes; that is,
    (8) $$\begin{array}{c} C=A\cup B=>C>=A;\;C>=B. \end{array}$$
   Consider the following.
    (i) If union class is the required class and its collection class as the advertised class, the match between them will be of type “subsume” as per (4).
    (ii) The Pellet reasoner adds collection classes *A* and *B* as the subclasses of *C*. Therefore, we have to remove collection classes from subclasses list of *C*.
     Now, if
      (9) $$\begin{array}{c} D=subclassOf\left(B\right)=>D>B, \end{array}$$
     from (8) and (9), *B* is smaller than both *C* and *D*, but we cannot comment on the relation between *C* and *D*. Therefore, we have to remove subclasses of *B* from the subclasses list of union class. That is, we have to remove sub classes of collection classes from the subclasses list of *C* generated by Pellet reasoner.
    (iii) The protégé editor interprets collection classes *A* and *B* as the equivalent classes of *C*. This can be dealt with using two ways: (i) we should remove the *A* and *B* from the equivalent classes list or (ii) we decide the sequence of match in a way that error prone equivalent classes list will not generate error prone match results. We match equivalent classes in the last step to avoid creating error prone match results.
     Resource discovery algorithm typically uses the Pellet-like reasoner, and usage of the protégé editor is not guaranteed. Therefore, instead of removing *A* and *B* classes from the equivalent classes list, a proper sequence to deal with error prone equivalent classes is more preferable, we believe.
  (b) In class hierarchy, if we have *C* as intersection class
    (10) $$\begin{array}{c} C=A\cap B=>C<=A;\;C<=B. \end{array}$$
   Consider the following.
    (i) If intersection class is the required class and collection classes are advertised classes, the match between them will be of type “plug-in” as per (5).
    (ii) The Pellet reasoner adds *C* as the subclass of collection classes. Therefore, we have to remove *C* from the subclasses of collection classes.
     Now, if
      (11) $$\begin{array}{c} D=subclassOf\left(C\right)=>C<D, \end{array}$$
     from (10) and (11) *C* is smaller than both *B* and *D*, but we cannot comment on the relation between *B* and *D*. Therefore, we have to remove subclasses of *C* from the subclasses list of collection classes generated by Pellet reasoner.
(2) The UnionIntersectionMatch algorithm. Our match algorithm matches the complement, union, intersection, sub-, equality, and equivalent classes in a strict sequence. The change in sequence may produce error prone match results, as discussed in The Logic subsection. We apply our subclasses calculation algorithm to the complement checking algorithm of [43]. It is displayed as isComplementOf function in the pseudo code section.

### 4.2. Pseudo Code of Our Algorithm

The pseudo code of our algorithms is as shown in Algorithms 1 and 2.

### 4.3. Theoretical Analysis of Our Algorithm

As our algorithms use simple loops, the worst case time complexity of our algorithm is *O*(*n*).

## 5. Methodology of Implementation

The implementation details of our proposed algorithm are as follows.

### 5.1. Tools Used

- Fedora (version 19): Fedora [44] is an open source application platform. It offers deployment of standalone applications, web servers, virtual servers, and so forth.
- Java Language (JDK1.7): Java [45, 46] language is simple, object oriented, robust, secured, architecture neutral, portable, interpreted, threaded, and dynamic, which enables high performance applications on multiple platforms in distributed environment.
- Jena semantic framework: jena [18] is a Java framework for developing semantic web applications. It provides good programmatic environment for RDF (Resource Description Framework), RDFS (RDF schema), and OWL. It includes SPARQL (The Simple Protocol and RDF Query Language) and a rule-based inference engine. The jena framework includes a RDF API that reads/writes RDF in RDF/XML, N3, and N-Triples.
- Pellet reasoner: the Pellet is an OWL 2 reasoner. It provides standard and cutting-edge reasoning services for OWL ontologies [20].
- Protégé: The protégé is an ontology editor. The support for Java and being open source make the protégé the popular reasoner [21, 22].
- Netbeans (version 8.0): The NetBeans IDE [47] is a platform independent and open source IDE for Java, PHP, Ruby and Ruby on Rails, Groovy and Grails and C/C++, JavaScript, and so forth languages. It enables developers to rapidly create web, enterprise, desktop, and mobile applications and distributed applications. Its support of suggesting files import on inserting jar files is very much useful to the new developer from the jar file.
- Apache web server: the apache web server [48] is an open source web server which is used here to host the ontology and service resources.

### 5.2. Implementation Details

Our implementation uses Linux Operating System, Java Programming Language, and Pellet reasoner. The implementation details are as follows.

- We add* named* union and intersection classes in the conference ontology [23] such that collection classes are not added as equivalent classes or subclasses of union class. They are as shown in Figure 4.
- We calculate subclasses of each class in ontology, using added union and intersection classes.
- We compare the results of calculating subclasses and match algorithm with our candidate reasoner, that is, the Pellet reasoner. Our algorithm uses the Pellet reasoner's output and rectifies that. Therefore, it should take more time to calculate subclasses.

**Figure 3:**
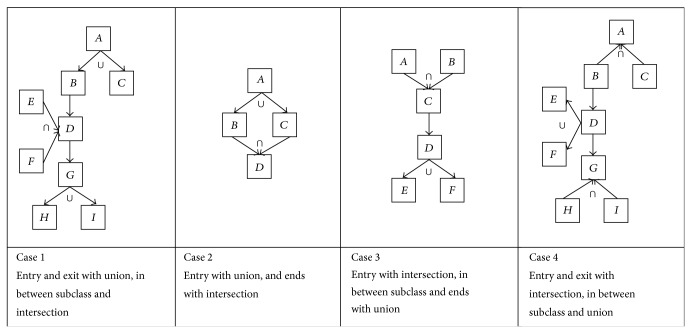
Cases of adding union and intersection classes.

We consider mainly four cases of adding mix of intersection and union classes based on their location, as shown in Figure 3.

- Case 1: union class as start and end classes and intersection class in between in class hierarchy.
- Case 2: union class as start and intersection class as end class in class hierarchy.
- Case 3: intersection class as start and union class as end in class hierarchy.
- Case 4: intersection class as start and end classes and union class in between in class hierarchy.

**Figure 4:**
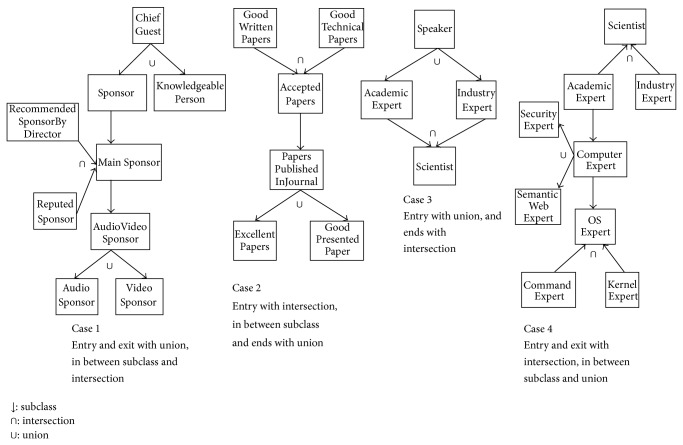
Cases of adding union and intersection classes in conference ontology.

We add the above mentioned for cases in the conference management system's ontology. It is shown in Figure 4.

### 5.3. Test Application

As the test application, we use the conference management system, because we believe the standard data set of conference ontology [23] is nowadays used to test the match algorithms.

### 5.4. Evaluation Metrics

We use precision ratio as the evaluation parameter.

## 6. Performance Results & Analysis

We have added the* named* union classes, intersection classes, and subclasses in an ontology with four different cases as shown in Figures 3 and 4. We compare the results of our subclasses calculating algorithm with those of the Pellet reasoner. From Tables 3, 4, 5, and 6, we can say, the execution time of our algorithm is more than that of the Pellet reasoner. The precision ratio of our subclass calculating algorithm increases from 70% to 100%. Though the execution time of our algorithm is more, it gives better precision ratio.* We can ignore investing few more milliseconds of time, to get ethically correct interpretations of union and intersection classes, with increased precision ratio.* The real world conference ontology with our added classes and cases results are as shown in Tables 7, 8, 9, and 10. We are able to get the corrected subclasses list using our algorithm.

**Figure 5:**
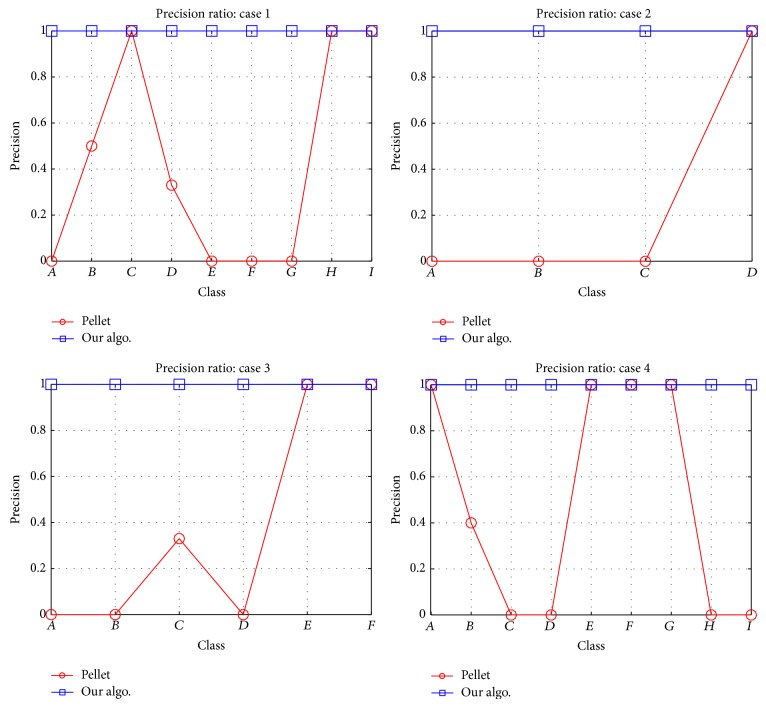
Subclasses calculation precision ratio by Pellet and our algo.: cases 1, 2, 3, and 4.

**Figure 6:**
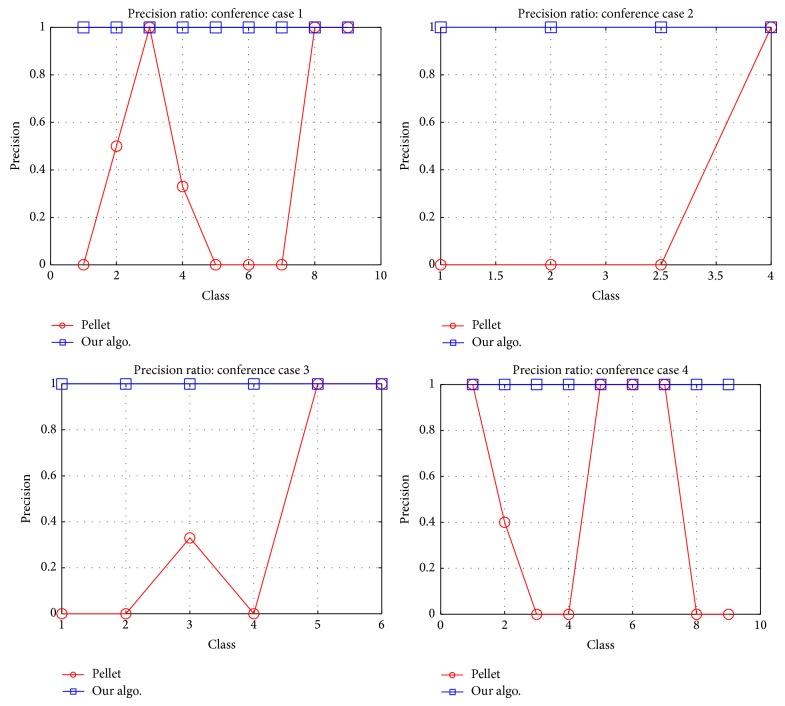
Subclasses calculation precision ratio by Pellet and our algo.: conference cases 1, 2, 3, and 4.

For general cases, our algorithm improves the precision ratio, as shown in Figure 5. For the conferences cases, the improved ratio is as shown in Figure 6. For UnionIntersectionMatch algorithm the performance results are as shown in Table 11. We can observe that the relation of union and intersection classes with their collection classes is not “exact match,” but rather subsume or plug-in match. The results obtained are different than those we get from the Pellet reasoner. The changes in the results are shown highlighted in Table 11.

## 7. Conclusion and Future Work

A resource discovery is a challenging task, because of the unlimited size of the semantic web. The ontologies available in literature* typically* contain anonymous union and intersection classes. The anonymous classes may not take part in the whole inference process of a reasoner. The candidate ontology editor, the protégé [21, 22], interprets the* named* union and its collection classes as equivalent classes. The candidate reasoner, namely, Pellet [20, 41, 49] adds the intersection class as subclass of its collection classes, whereas it adds union class as superclass of its collection classes. Therefore, the relations between collection classes with their intersection and union class are error prone. In addition, the subclasses list inferred by the Pellet also can be error prone. Therefore, in this paper we have proposed algorithms to (a) relate intersection and union classes correctly to their collection classes, (b) calculate correct subclasses list of any class of ontology, wherein the class hierarchy contains intersection and union classes, and (c) match the required and advertised classes using proper sequence of the above two steps with complement classes added. We have compared our subclasses calculation algorithm results with those of our candidate reasoner, that is, the Pellet reasoner. Though the execution time of our subclasses calculation algorithm is more than that of the Pellet reasoner, we get improved precision ratio from 70% to 100% for our ontology cases. The UnionIntersectionMatch algorithm has different match results than those of the Pellet reasoner. Our match algorithm must follow the steps in the specified sequence of (complement, union, intersection, sub-, equality, and equivalent classes) ONLY, or else it may produce error prone results. To the best of our knowledge, ours is a unique and novel way to relate ontology classes.

As a future work, one can find proper subclasses of anonymous union and intersection classes of existing ontologies. One can propose subproperties inclusion in the algorithm. One can use our finally improved algorithm with content based similarity measure of Signature Quadratic Form Distance (SQFD) [50] to find alignment between two ontologies. Our algorithm, if used in ontology alignment algorithm, will improve precision ratio of resource discovery, we believe.

## Figures and Tables

**Algorithm 1:**
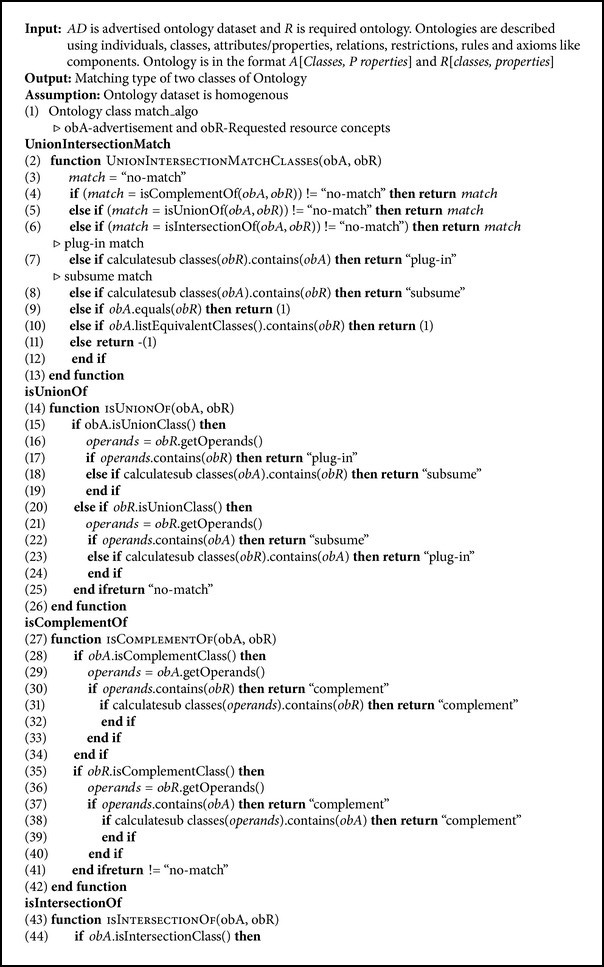
Pseudo code of our UnionIntersectionMatch algorithm.

**Algorithm 2:**
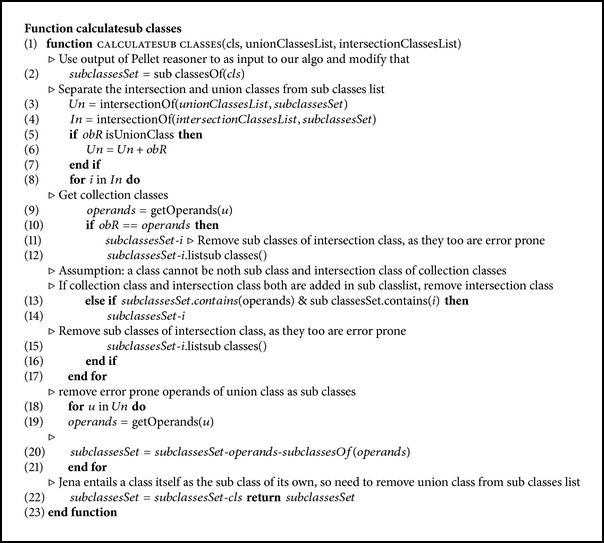
Pseudo code of our Calculated subclasses algorithm.

**Table 1:** Comparison of jena query engine and Pellet query engine.

Criteria	Jena query engine	Pellet query engine
Number of queries dealt with	RDF triple based, so it works one triple at a time	It considers the entire conjunctive query

Speed of results	Query optimizations are not accessible. So no speedup based on that is not available.	Query optimizations are accessible and perform optimizations too and therefore can speed up the query result

Blank nodes/anonymous resources	For anonymous resource, it will not produce result	For anonymous resource, it will produce result

**Table 2:** Comparison of interpretation of union and intersection classes by protégé vs. Pellet.

Class	Interpretation by	
	Protégé ontology editor	Pellet reasoner
Intersection class	It adds intersection class as error prone of collection classes	It interprets intersection class as error prone of collection classes

Union class	It treats union class as equivalent of collection classes	It adds union class as super class of collection classes

**Table 3:** Calculating list of sub classes: case 1.

Number	Class	Subclasses by Pellet	Time by Pellet (ms)	Subclasses by our algo.	Time by our algo. (ms)	Precision by Pellet	Precision by our algo.
1	A	[B, C, D, G, H, and I]	1	[]	70	0.00	1.00
2	B	[D, G, H, and I]	1	[D and G]	35	0.50	1.00
3	C	[]	0	[]	0	1.00	1.00
4	D	[G, H, and I]	1	[G]	32	0.33	1.00
5	E	[D, G, H, and I]	1	[]	86	0.00	1.00
6	F	[D, G, H, and I]	1	[]	62	0.00	1.00
7	G	[H and I]	40	[]	85	0.00	1.00
8	H	[]	0	[]	0	1.00	1.00
9	I	[]	0	[]	0	1.00	1.00

**Table 4:** Calculating list of sub classes: case 2.

Number	Class	Subclasses by Pellet	Time by Pellet (ms)	Subclasses by our algo.	Time by our algo. (ms)	Precision by Pellet	Precision by our algo.
1	A	[B, C, and D]	9	[]	45	0.00	1.00
2	B	[D]	29	[]	49	0.00	1.00
3	C	[D]	0	[]	18	0.00	1.00
4	D	[]	0	[]	1	1.00	1.00

**Table 5:** Calculating list of subclasses: case 3.

Number	Class	Subclasses by Pellet	Time by Pellet (ms)	Subclasses by our algo.	Time by our algo. (ms)	Precision by Pellet	Precision by our algo.
1	A	[C, D, E, and F]	35	[]	83	0.00	1.00
2	B	[C, D, E, and F]	1	[]	44	0.00	1.00
3	C	[D, E, and F]	1	[D]	24	0.33	1.00
4	D	[E and F]	1	[]	19	0.00	1.00
5	E	[]	0	[]	0	1.00	1.00
6	F	[]	0	[]	0	1.00	1.00

**Table 6:** Calculating list of subclasses: case 4.

Number	Class	Subclasses by Pellet	Time by Pellet (ms)	Subclasses by our algo.	Time by our algo. (ms)	Precision by Pellet	Precision by our algo.
1	A	[]	0	[]	1	1.00	1.00
2	B	[A, D, E, F, and G]	1	[D and G]	94	0.40	1.00
3	C	[A]	0	[]	21	0.00	1.00
4	D	[E, F, and G]	1	[G]	35	0.00	1.00
5	E	[]	15	[]	15	1.00	1.00
6	F	[]	1	[]	1	1.00	1.00
7	G	[]	53	[]	55	1.00	1.00
8	H	[G]	0	[]	22	0.00	1.00
9	I	[G]	0	[]	16	0.00	1.00

**Table 7:** Conference cases subclass calculation results: case 1.

Number	Class	Pellet subclasses	Our algo. subclasses
1	Chief guest	[Sponsor, KnowledgeablePerson, MainSponsor, AudioVideoSponsor, AudioSponsor, and VideoSponsor]	[]

2	Sponsor	[MainSponsor, AudioVideoSponsor, AudioSponsor, and VideoSponsor]	[MainSponsor and AudioVideoSponsor]

3	KnowledgeablePerson	[]	[]

4	MainSponsor	[AudioVideoSponsor, AudioSponsor, and VideoSponsor]	[AudioVideoSponsor]

5	RecommendedSponsorByDirector	[MainSponsor, AudioVideoSponsor, AudioSponsor, and VideoSponsor]	[]

6	ReputedSponsor	[MainSponsor, AudioVideoSponsor, AudioSponsor, and VideoSponsor]	[]

7	AudioVideoSponsor	[AudioSponsor and VideoSponsor]	[]

8	AudioSponsor	[]	[]

9	VideoSponsor	[]	[]

**Table 8:** Conference cases subclass calculation results: case 2.

Number	Class	Subclasses by Pellet	Subclasses by our algo.	Precision by Pellet	Precision by our algo.
1	Speaker	[AcademicExpert, IndustryExpert, and Scientist]	[]	0.00	1.00

2	AcademicExpert	[Scientist]	[]	0.00	1.00

3	IndustryExpert	[Scientist]	[]	0.00	1.00

4	Scientist	[]	[]	1.00	1.00

**Table 9:** Conference cases subclass calculation results: case 3.

Number	Class	Subclasses by Pellet	Subclasses by our algo.	Precision by Pellet	Precision by our algo.
1	WellWrittenPapers	[AcceptedPapers, PapersPublishedInJournal, ExcellentPapers, and WellPresentedPaper]	[]	0.00	1.00

2	GoodTechnicalPapers	[AcceptedPapers, PapersPublishedInJournal, ExcellentPapers, WellPresentedPaper]	[]	0.00	1.00

3	AcceptedPapers	[PapersPublishedInJournal, ExcellentPapers, and WellPresentedPaper]	[PapersPublishedInJournal]	0.33	1.00

4	PapersPublishedInJournal	[ExcellentPapers and WellPresentedPaper]	[]	0.00	1.00

5	ExcellentPapers	[]	[]	1.00	1.00

6	WellPresentedPaper	[]	[]	1.00	1.00

**Table 10:** Conference cases subclass calculation results: case 4.

Number	Class	Subclasses by Pellet	Subclasses by our algo.	Precision by Pellet	Precision by our algo.
1	Scientist	[]	[]	1.00	1.00

2	AcademicExpert	[Scientist, ComputerExpert, SecurityExpert, SemanticWebExpert, and OSExpert]	[ComputerExpert and OSExpert]	0.40	1.00

3	IndustryExpert	[Scientist]	[]	0.00	1.00

4	ComputerExpert	[SecurityExpert, SemanticWebExpert, and OSExpert]	[OSExpert]	0.33	1.00

5	SecurityExpert	[]	[]	1.00	1.00

6	SemanticWebExpert	[]	[]	1.00	1.00

7	OSExpert	[]	[]	1.00	1.00

8	CommandExpert	[OSExpert]	[]	0.00	1.00

9	KernelExpert	[OSExpert]	[]	0.00	1.00

**Table 11:** UnionIntersectionMatch algorithm's improved results.

obA	obR	Relation of obR to obA	Match by Pellet	Match by our algo.
AcceptedPaper	RejectedPaper	Complement	Complement	Complement
AcademicExpert	ComputerExpert	subclass	Subsume	Subsume
ComputerExpert	AcademicExpert	superclass	Plug-in	Plug-in
ChiefGuest	Sponsor	Operand of union Class	**Subsume **	**Plug-in **
AcceptedPapers	WellWrittenPapers	Operand of intersection Class	**Plug-in **	**Subsume **
ChiefGuest	MainSponsor	subclass of operand of union Class	**Subsume **	**No match **
Scientist	ComputerExpert	subclass of operand of intersection Class	No match	No match
